# Identification of ferroptosis-related gene signatures in temporal lobe epilepsy with hippocampal sclerosis

**DOI:** 10.3389/fnins.2025.1530182

**Published:** 2025-04-02

**Authors:** Fan Gao, Jinzi Li

**Affiliations:** Department of Pediatrics, Yanbian University Hospital, Yanji, China

**Keywords:** ferroptosis, temporal lobe epilepsy with hippocampal sclerosis, biomarkers, differentially expressed genes, receiver operating characteristic

## Abstract

**Background:**

Ferroptosis is a form of regulated cell death that damages neurons in the central nervous system. In this study, we aimed to construct ferroptosis-related gene signatures in temporal lobe epilepsy with hippocampal sclerosis (TLE-HS) and explore their diagnostic role in TLE-HS.

**Methods:**

The GSE205661 dataset was acquired for training purposes, while the GSE71058 was obtained to serve as the validation dataset. Subsequently, ferroptosis-related differentially expressed genes (FR-DEGs) in TLE-HS were further analyzed. We used weighed gene co-expression network analysis (WGCNA) algorithm, single-factor logistic regression analysis, and LASSO algorithm to screen characteristic FR-DEGs. Then, the receiver operating characteristic (ROC) was used to evaluate the value of these characteristic genes in disease diagnosis. Finally, a long non-coding RNA (lncRNA)–microRNA (miRNA)–messenger RNA (mRNA) network was constructed.

**Results:**

We identified 141 FR-DEGs in TLE-HS, and these genes were enriched in T-cell activation involved in immune response and signaling pathways related to lipids and atherosclerosis. Further WGCNA was performed to select 47 overlapping FR-DEGs, which were significantly enriched in 13 biological processes and 14 Kyoto Encyclopedia of Genes and Genomes (KEGG) pathways, including the negative regulation of apoptotic process and ferroptosis. Four genes, namely *PDK4*, *SMPD1*, *GPT2*, and *METTL14*, were identified as signature genes in TLE-HS. Moreover, the ROC derived from the four genes in GSE205661 and GSE71058 for predicting TLE-HS had an area under the curve (AUC) of 0.988 and 0.929, respectively. Furthermore, the lncRNA–miRNA–mRNA network constructed from the 4 FR-DEGs consisted of 5 lncRNAs and 14 miRNAs. The signatures based on four FR-DEGs were found to be a strong predictor of TLE-HS, and they may represent valuable therapeutic targets for TLE-HS.

## 1 Introduction

Temporal lobe epilepsy (TLE) is one of the primary types of focal epilepsy, characterized by aberrant neuronal discharges or impairments in the temporal lobe cortex. The temporal lobe’s high susceptibility to epileptic seizures makes TLE one of the most prominent epilepsy syndromes (Engel, 2016). Currently, surgical resection is the primary treatment strategy (Jones and Cascino, 2016), and hippocampal sclerosis (HS) represents the most common pathology found in adult epilepsy surgery, accounting for over 50–80% of TLE cases (Allone et al., 2017). Although various techniques have emerged as auxiliary tools for diagnosing and treating TLE (Jiao et al., 2025), the condition is mainly diagnosed based on a history of characteristic partial seizure symptoms in the clinic (He et al., 2022). In the early stage of TLE, limited signatures could be detected. To reduce the brain damage caused by the subsequent seizure of epilepsy, there is an urgent need to investigate more valuable diagnostic tools for TLE.

In recent years, bioinformatics analysis has investigated some valuable biomarkers for TLE and offered new insights into the process of TLE development. For example, He et al. demonstrated that TIMP1 is the most significant inflammation-related gene associated with TLE, and its expression is downregulated in both epilepsy patients and experimental mice (He et al., 2023). Chen et al. identified six feature genes associated with epilepsy and confirmed their valuable diagnostic role in TLE (Chen S. et al., 2023). Notably, ferroptosis, a newly identified type of regulatory cell death caused by the accumulation of excessive iron ions, could lead to the lipid damage mediated by the generation of reactive oxygen species (ROS) (Zheng et al., 2024). In the epileptic focus, oxidative stress and iron overload are proposed as common pathological characteristics. During high-intensity brain activity, lipid peroxidation levels can be elevated, which is triggered by the oxidative stress products and excessive free fatty acids. A recent study by Chen et al. demonstrated abnormal lipid accumulation in TLE (Chen Z. P. et al., 2023). Meanwhile, additional evidence confirmed the association between central nervous system neurons damage and the accumulation and imbalance of free irons (Levi et al., 2024). Thus, ferroptosis could provide effective diagnostic targets for TLE-HS. Although some researchers tried to explore ferroptosis-related activity in TLE-HS, the exploration of diagnostic biomarkers related to ferroptosis in TLE-HS remains limited.

Consequently, GSE205661 and GSE71058 were downloaded as the training and validation datasets, respectively. Then, we screened ferroptosis related differentially expressed genes (FR-DEGs) in TLE-HS. Furthermore, the diagnostic model based on characteristic FR-DEGs was constructed to explore valuable ferroptosis-related targets and further analyze the molecular mechanisms underlying the progression of TLE-HS.

## 2 Materials and methods

### 2.1 Microarray data of TLE-HS

The transcriptional profiles of TLE-HS were downloaded from the National Center for Biotechnology Information Gene Expression Omnibus (NCBI GEO) (Barrett et al., 2013). The dataset was selected based on the keyword “temporal lobe epilepsy with hippocampal sclerosis (TLE-HS).” GSE205661 (Wang et al., 2022) was obtained as training dataset because it aligns with the TLE-HS disease type and provides comprehensive expression data for long non-coding RNAs (lncRNAs), microRNAs (miRNAs), and mRNAs. This dataset includes 15 samples, comprising six TLE-HS patients and nine controls.

Meanwhile, the dataset GSE71058 (Griffin et al., 2016) was downloaded as a validation dataset. This dataset contains 22 hippocampal tissue samples, including eight TLE-HS cases and 14 controls, and aligns GSE205661 in terms of disease type (TLE-HS). Furthermore, both datasets are derived from hippocampal tissue, ensuring consistency in tissue source and enhancing the comparability between the training and validation datasets.

### 2.2 Screening of deferentially expressed RNA (DERs)

Based on the sample information, Limma version 3.34.0 (Ritchie et al., 2015)^1^ (Bioconductor project) in R4.3.1 language was used to screen DERs in the TLE-HS vs. control, including lncRNA, miRNA, and mRNA. False discovery rate (FDR) < 0.05 and |log_2_ fold change (FC)| > 0.5 were set as the thresholds for DERs.

Ferroptosis related genes (FRGs) were downloaded from FerrDb database^2^ (Zhou et al., 2023), including driver, suppressor, marker, inhibitor, inducer, and unclassified. Then, DERs were compared to FRGs, and the overlapping genes were retained, which were defined as FR-DEGs. Gene Ontology (GO) biology process and Kyoto Encyclopedia of Genes and Genomes (KEGG) signal pathway enrichment analysis for FR-DEGs were performed by DAVID version 6.8 (Huang da et al., 2009a,b) (National Institutes of Health).^3^ The *p*-value less than 0.05 was selected as the threshold of enrichment significance.

### 2.3 Selecting of TLE-HS related genes based on weighed gene co-expression network analysis algorithm

WGCNA is a bioinformatics algorithm designed to construct co-expression networks and further identifies disease-associated modules or potential therapeutic targets. Based on all the genes detected in the GSE205661 dataset, WGCNA version 1.61 package (Bioconductor Project) in R4.3.1 language (Langfelder and Horvath, 2008)^4^ was used to filter modules associated with disease states. The threshold for module partitioning was set as follows: The module set contains at least 200 genes with cutHeight set at 0.995. The modules with an absolute correlation to disease status greater than 0.3, along with the genes involved in those modules, were retained as disease-related candidate genes. Finally, the genes in the retained disease-related modules were compared with FR-DEGs, and the overlapping parts were retained as disease-related FR-DEGs candidate genes.

### 2.4 Construction of network based on disease-related FR-DEGs

Based on the lncRNA–miRNA pairs obtained from DIANA-LncBase^5^ (Paraskevopoulou et al., 2016), the regulatory relationship between differentially expressed lncRNA and miRNA was selected, and the pairs with the miRNA target gene score (miTG-score) higher than 0.6 were retained. Then, the miRWalk 3.0 database (Li et al., 2020)^6^ was used to search for the target genes of miRNAs associated with lncRNA, and these target genes were compared with the disease-related FR-DEGs candidate genes. The overlapping parts were retained as regulated FR-DEGs. Finally, the regulatory network of FR-DEGs candidate genes was visualized using Cytoscape version 3.9.0 (Shannon et al., 2003)^7^ (Cytoscape Consortium) through comprehensively analyzing lncRNA–miRNA and miRNA-target genes relationships.

### 2.5 Construction of a diagnostic model based on disease-related FR-DEGs

#### 2.5.1 Single-factor logistic regression analysis

The single-factor logistic regression analysis was performed using the RMS R4.3.1 version 6.3–0^8^ (Pan et al., 2021) based on the expression level of FR-DEGs. FR-DEGs with *p* < 0.05 would be retained.

#### 2.5.2 Selection of optimal FR-DEGs combinations

The optimal FR-DEGs combinations were further selected using the least absolute shrinkage and selection operator (LASSO) algorithm based on the lars packages version 1.2 (Wang and Liu, 2015)^9^ (Comprehensive R Archive Network) in R4.3.1 language.

#### 2.5.3 Construction of diagnostic model

The disease diagnosis classifier based on FR-DEGs was constructed using support vector machine (SVM) method in R4.3.1 e1071 version 1.6–8^10^ (Comprehensive R Archive Network) based on data from the training set. Then, the effectiveness evaluation of the classifier based on GSE205661 training dataset was analyzed by the receiver operating characteristic (ROC) curve method in R3.6.1 pROC version 1.12.1 (Robin et al., 2011) (Comprehensive R Archive Network).^11^ The expression levels of important FR-DEGs and the efficacy of the diagnostic model were verified by data from the validation dataset.

Then, multiple decision curve analysis was performed to analyze the net return of each gene on the outcome of the sample using R4.3.1 language rmda package version 1.6 (Harrell et al., 1996) (Comprehensive R Archive Network),^12^ and further, the influence of different genes on the sample species was also compared. Finally, the regulatory network of characteristic FR-DEGs was constructed, from which, important lncRNAs and miRNAs associated with FR-DEGs were analyzed.

### 2.6 Animals

Male Sprague Dawley rats (220–250 g, 6–8 weeks old) were obtained from Shanghai SLAC Animal Co., Shanghai, China. The rats were randomly divided into a model group and a control group, each comprising 15 rats. They were housed in a specific pathogen-free facility at a constant temperature of 24 ± 1°C with a 12-h light/dark cycle, and had unlimited access to food and water. All experimental procedures were conducted in accordance with relevant guidelines and regulations. The animal experiments adhered to the ARRIVE guidelines and were approved by the Biomedical Ethics Committee of Yanbian University (approval no: 2023228).

As previously described, a Lithium-Pilocarpine-Induced Status Epilepticus (SE) model was established (Eslami et al., 2022). In summary, the rats in the model group received an intraperitoneal injection of 125 mg/kg lithium chloride. Approximately 18 h later, the rats were subcutaneously administered 1-mg/kg methylscopolamine (MilliporeSigma, Burlington, MA, United States) to mitigate the undesired peripheral effects of pilocarpine. SE was induced approximately 30 min later by an intraperitoneal injection of 20-mg/kg pilocarpine (Abcam). The control group was given lithium chloride and saline instead of pilocarpine. Seizures typically commenced 10–30 min following pilocarpine administration. The severity of seizures was assessed using a modified Racine scale (Racine, 1972). According to the Racine scale, seizure intensity was classified as follows: Grade I: immobility, eyes closed, and facial clonus; grade II: head nodding and more severe facial clonus; grade III: clonus of one forelimb; grade IV: rearing with bilateral forelimb clonus; and grade V: generalized tonic–clonic seizures. The rats demonstrating grade IV or V on the Racine scale were considered to have successfully modeled SE. If SE persisted for over 1 h, 10% chloral hydrate was administered intraperitoneally to terminate SE. The rats were deeply anesthetized using 4% chloral hydrate, euthanized through cervical dislocation, and then their brain tissues were extracted.

### 2.7 Reverse transcription-quantitative polymerase chain reaction

The total RNA was isolated from cells using TRIzol reagent (Thermo Fisher Scientific, Waltham, MA, USA) and subsequently reverse transcribed with the PrimeScript RT reagent Kit (TaKaRa, Kyoto, Japan). For RT-qPCR analysis, the SYBR Green Quantitative RT-PCR Master Mix kit (Toyobo Co., Ltd., Osaka, Japan) was employed. Relative mRNA expression levels were determined using the 2-ΔΔCT method, with GAPDH serving as the internal reference. The primer sequences are listed in Table 1.

**Table 1:** The primer sequences used in this study.

Gene	Primer (5′-3′)
PDK4-rF	CAAGATTTCTGACCGAGGAG
PDK4-rR	CTGATAATGTTTGAAGGCTGAC
SMPD1-rF	CCAATGTGGCACGAGTAGGC
SMPD1-rR	TTCGGCACTGATGGCAAAGA
GPT2-rF	TCTTTGTGCCTTGATGTTCG
GPT2-rR	AAGCAGGTTGACTACTTTGGTG
METTL14-rF	GCAGAAACCTACGCGTCCTA
METTL14-rR	CACCACGGTCAGACTTGGAT
GAPDH-rF	AGACAGCCGCATCTTCTTGT
GAPDH-rR	CTTGCCGTGGGTAGAGTCAT

### 2.8 Statistical analysis

The statistical analyses were conducted using GraphPad Prism software version 9 (GraphPad Software, Boston, MA, United States). The data are presented as mean ± standard deviation (SD). The two groups were compared using Student’s *t*-test, with a significance threshold set at *p* < 0.05.

## 3 Results

### 3.1 141 FR-DEGs in TLE-HS were selected

**Figure 1:**
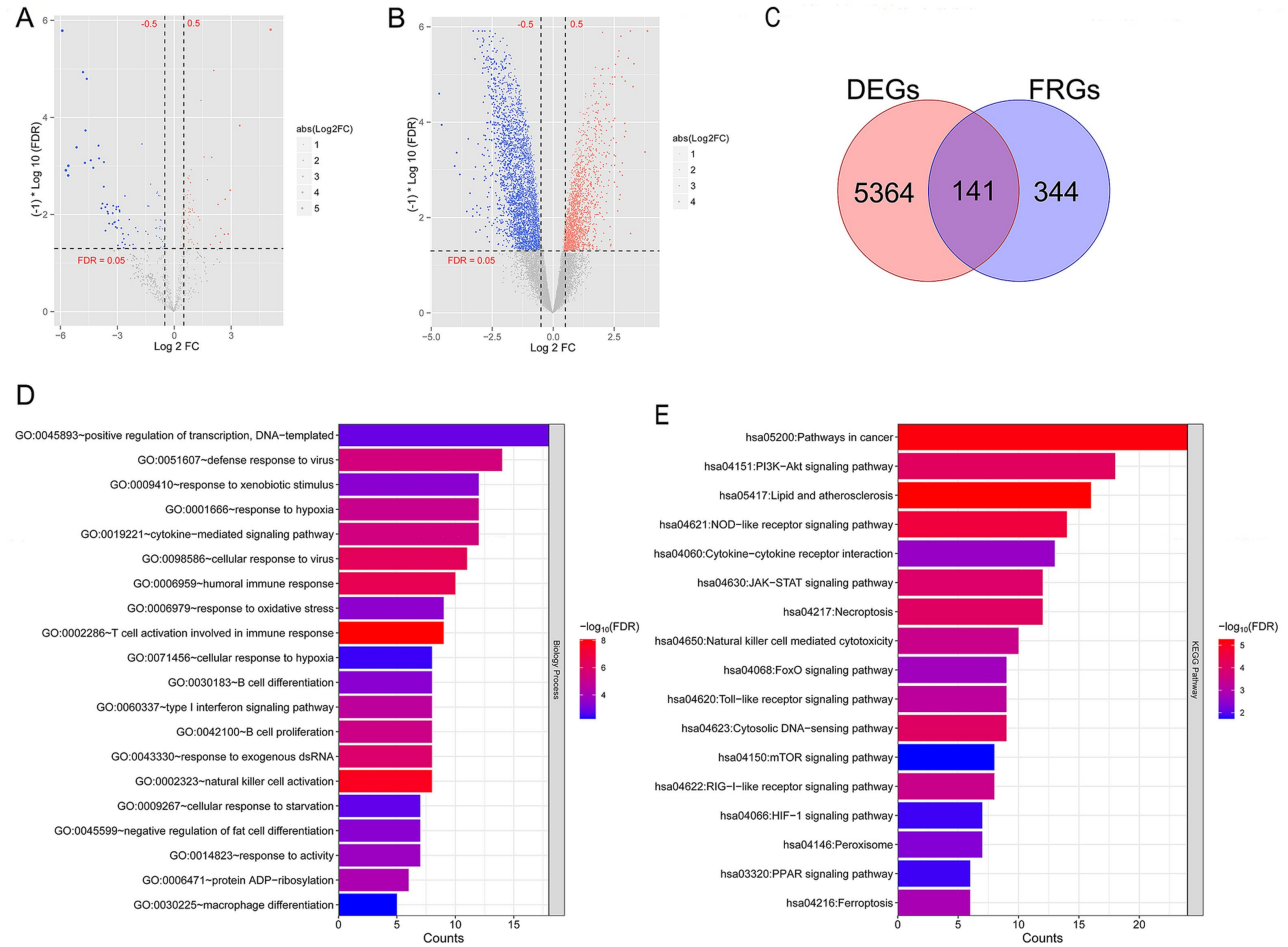
A total of 141 ferroptosis related differentially expressed genes (FR-DEGs) in temporal lobe epilepsy with hippocampal sclerosis (TLE-HS) were selected, and they were mainly enriched in T cell activation involved in immune response and signaling pathways related with lipid and atherosclerosis. **(A)** Volcano map of microRNA (miRNA); **(B)** volcano map of long non-coding RNAs (lncRNA) and mRNA; the red and blue dots represent significantly upregulated and downregulated DEGs, the horizontal dashed line represents False discovery rate (FDR) < 0.05, and the vertical dashed line represents|log_2_ fold change (FC)| > 0.5. **(C)** The Venn diagram of FR-DEGs; **(D)** Gene Ontology (GO) biological process enriched by FR-DEGs; and **(E)** Kyoto Encyclopedia of Genes and Genomes (KEGG) pathways enriched by FR-DEGs. The *p*-value below 0.05 was selected as the threshold of enrichment significance. The horizontal axis represents the number of genes, the vertical axis represents the item name, and the color represents significance.

A total of 5,505 mRNAs (3,426 downregulated and 2,079 upregulated), 9 lncRNAs (all downregulated), and 145 miRNAs (76 downregulated and 69 upregulated) in TLE-HS vs. control were screened. Volcano maps of differentially expressed miRNAs and differentially expressed lncRNA and mRNA are shown in Figures 1A,B, respectively. Meanwhile, 485 FRGs were obtained based on the FerrDb database. Then, we compared 485 FRGs and 5,505 mRNAs. Finally, 141 FR-DEGs were screened (Figure 1C).

Further functional enrichment analysis showed 40 biological processes out of GO items (Figure 1D), and 17 KEGG pathways (Figure 1E) were significantly enriched by 141 FR-DEGs (*p* < 0.05). Notably, these genes were mainly enriched in T-cell activation, which is involved in immune response and signaling pathways related to lipids and atherosclerosis.

### 3.2 47 disease-related FR-DEGs were screened

**Figure 2:**
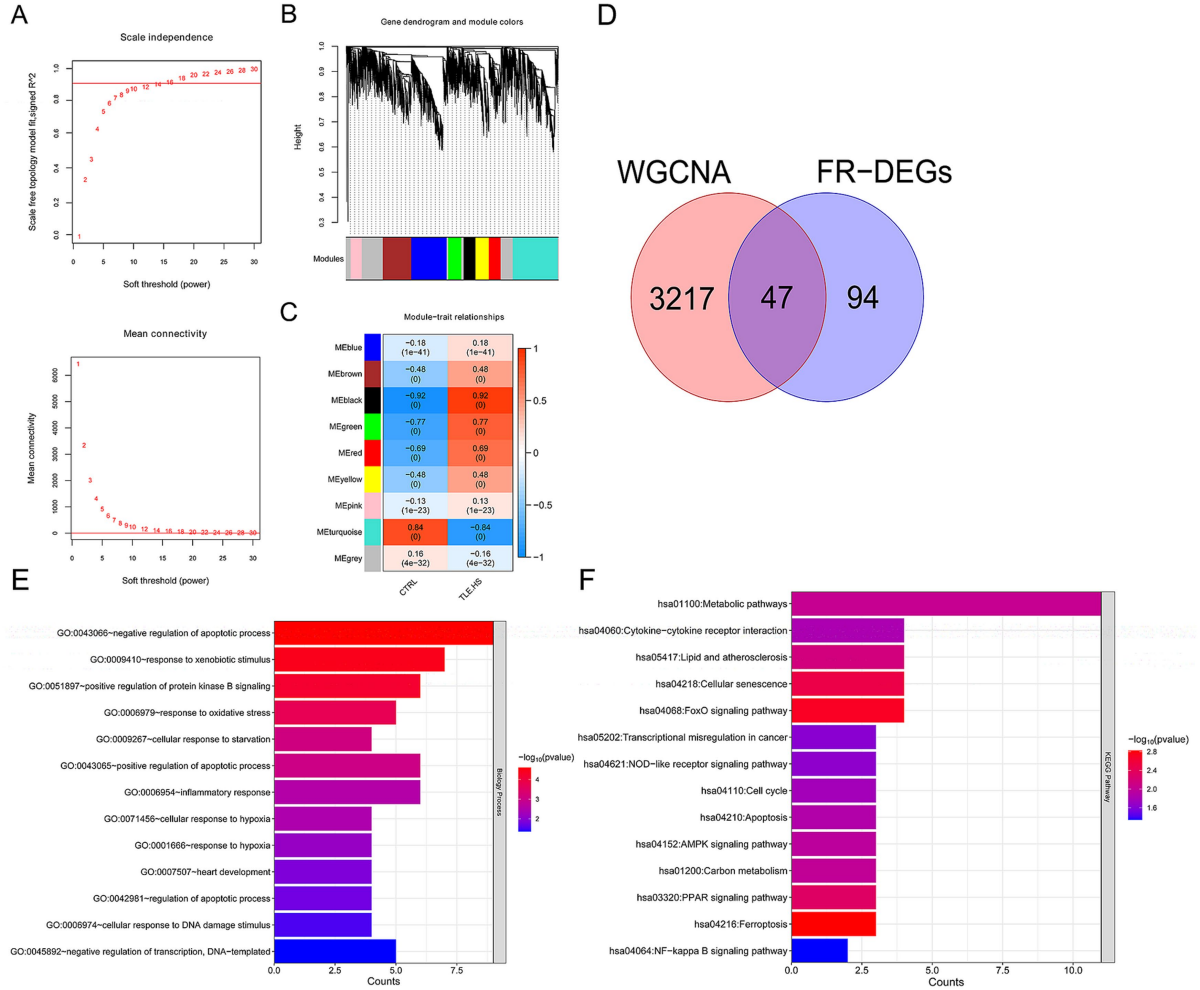
A total of 47 disease-related ferroptosis related differentially expressed genes (FR-DEGs) were screened, and they were enriched in 13 Gene Ontology (GO) biological processes and 14 Kyoto Encyclopedia of Genes and Genomes (KEGG) pathways. **(A)** Above: selection diagram of the adjacency matrix weight parameter power. The horizontal axis represents the weight parameter power, and the vertical axis represents the square of the correlation coefficients between log(k) and log(p(k)) in the corresponding network. The red line represents the standard line where the square value of the correlation coefficient reaches 0.9. Below: A schematic diagram of the average connectivity of genes under different power parameters. The red line represents the value of the average connectivity of network nodes (1) under the weight parameter power of the adjacency matrix in the left figure; **(B)** module partitioning tree diagram with each color representing different module; **(C)** module-trait correlation heatmap; **(D)** The Venn diagram of WGCNA selected genes and FR-DEGs. **(E)** GO biological process enriched by FR-DEGs; and **(F)** Kyoto Encyclopedia of Genes and Genomes (KEGG) pathways enriched by FR-DEGs. The *p-*value below 0.05 was selected as the threshold of enrichment significance. The horizontal axis represents the number of genes, the vertical axis represents the item name, and the color represents significance.

To satisfy the premise of scale-free network distribution, we explored the value of the adjacency matrix weight parameter power based on all genes detected in the GSE205661 dataset. As shown in Figure 2A, the value of power was selected when the square value of correlation coefficient reaches 0.9 for the first time, that is, power = 16. Currently, the average node connectivity of the constructed co-expression network is 1, which fully aligns with the characteristics of the small-world network. Then, the coefficient of divergence between gene points was calculated, and a systematic clustering tree was obtained. Nine modules were obtained when we set the minimum number of genes in each module as 200 and the cutHeight as 0.995 (Figure 2B). As shown in Figure 2C, six modules (black, brown, green, red, turquoise, and yellow) with absolute correlation with disease status higher than 0.3 were screened, which included 3,264 genes and recommended as disease-related candidate genes.

Then, these 3,264 genes were compared with 141 FR-DEGs, and 47 overlapping genes were obtained (Figure 2D). Furthermore, these 47 overlapping genes were significantly enriched in 13 biological processes (Figure 2E) and 14 KEGG pathways (Figure 2F), including negative regulation of apoptotic process and ferroptosis (*p* < 0.05).

### 3.3 Construction and verification of a diagnostic model based on disease-related FR-DEGs

**Figure 3:**
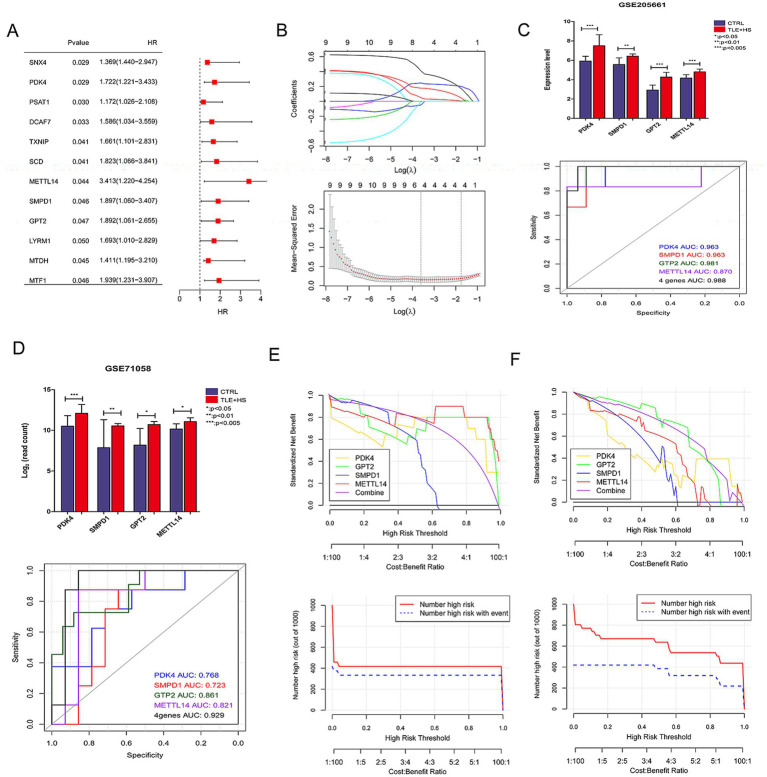
The screening of characteristic ferroptosis related differentially expressed genes (FR-DEGs) in temporal lobe epilepsy with hippocampal sclerosis (TLE-HS) and diagnostic model based on disease-related FR-DEGs genes. **(A)** Single-factor logistic regression of FR-DEGs, FR-DEGs with *p* < 0.05 would be retained; **(B)** the least absolute shrinkage and selection operator (LASSO) filtering parameter display diagram; **(C)** the expression levels of four FR-DEGs in TLE-HS and controls and receiver operating characteristic (ROC) based on the four FR-DEGs in GSE205661; **(D)** the expression levels of four FR-DEGs in TLE-HS and controls and ROC based on the four FR-DEGs in GSE71058; **(E)** decision line diagram of GSE205661; and **(F)** decision line diagram of GSE71058.

The initial screening through univariate logistic regression analysis identified 12 FR-DEGs significantly associated with TLE-HS (*p* < 0.05, Figure 3A). The subsequent analysis using the LASSO algorithm refined this to four genes: *PDK4*, *SMPD1*, *GPT2*, and *METTL14*, which were identified as optimal characteristic genes (Figure 3B).

The expression profiles of *PDK4*, *SMPD1*, *GPT2*, and *METTL14* were analyzed in GSE205661 and GSE71058 datasets using the limma, as depicted in Figures 3C,D. In both datasets, the expression levels of these four genes were significantly elevated in TLE-HS patients compared to controls (*p* < 0.05). Subsequently, the expression of these genes was validated in an animal model. The results indicated that the expression of *PDK4*, *SMPD1*, and *METTL14* was significantly increased in the model group compared to the control group (*p* < 0.05, Supplementary Figure S1). At the same time, *GPT2* showed no significant difference between the two groups (*p* > 0.05, Supplementary Figure S1). The ROC analysis demonstrated that these four genes provided strong predictive power for TLE-HS, with an area under the curve (AUC) of 0.988 in GSE205661 and 0.929 in GSE71058, highlighting their potential as biomarkers for this condition.

Subsequently, the decision curve analysis was performed to evaluate the clinical utility of models based on individual and combined gene expressions in both training and validation datasets. Figures 3E,F illustrate that the model combining all four genes offers the highest net benefit in both GSE205661 and GSE71058, indicating a superior diagnostic advantage.

### 3.4 LncRNA–miRNA–mRNA network

**Figure 4:**
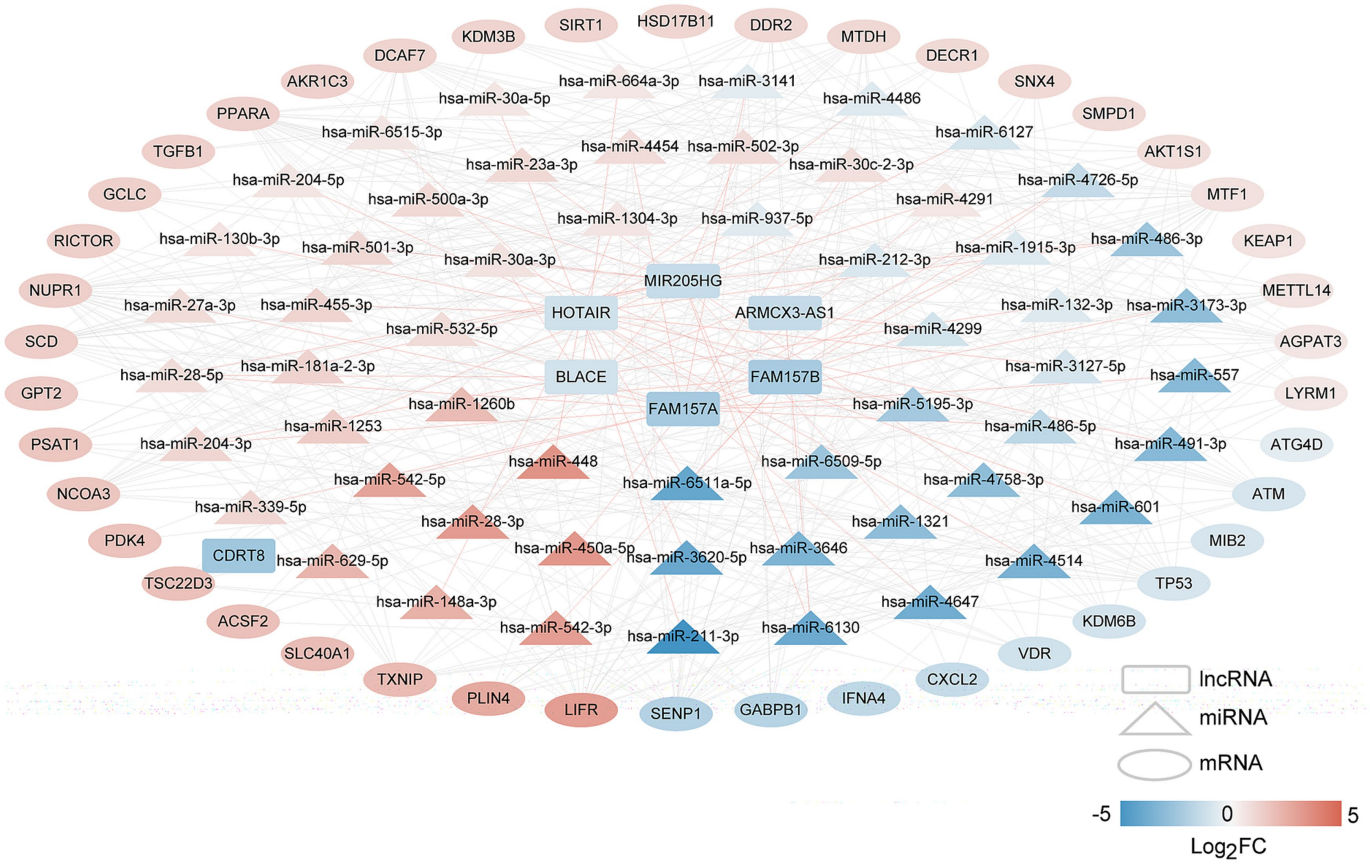
The long non-coding RNAs (lncRNAs)–microRNAs (miRNAs)–mRNAs network of disease-related FR-DEGs. Squares, triangles, and circles represent lncRNAs, miRNAs, and mRNAs, respectively, with colors indicating the degree of difference. The red connecting line represents lncRNAs–miRNAs, and the gray connecting line represents miRNAs–mRNAs.

We constructed a lncRNA–miRNA–mRNA network to elucidate the regulatory interactions within the biological system. Initially, lncRNA–miRNA pairs with a miTG-score exceeding 0.6 were identified, producing 71 pairs comprising seven lncRNAs and 57 miRNAs. Then, the genes targeted by 57 miRNAs were screened, and the target genes were compared with disease-related FR-DEGs genes. The overlapping genes were retained as regulated FR-DEGs, and 394 miRNA-mRNA link pairs were obtained. Based on these interactions, we constructed a comprehensive lncRNA–miRNA–mRNA network (Figure 4), which includes 7 lncRNAs, 57 miRNAs, and 42 mRNAs.

**Figure 5:**
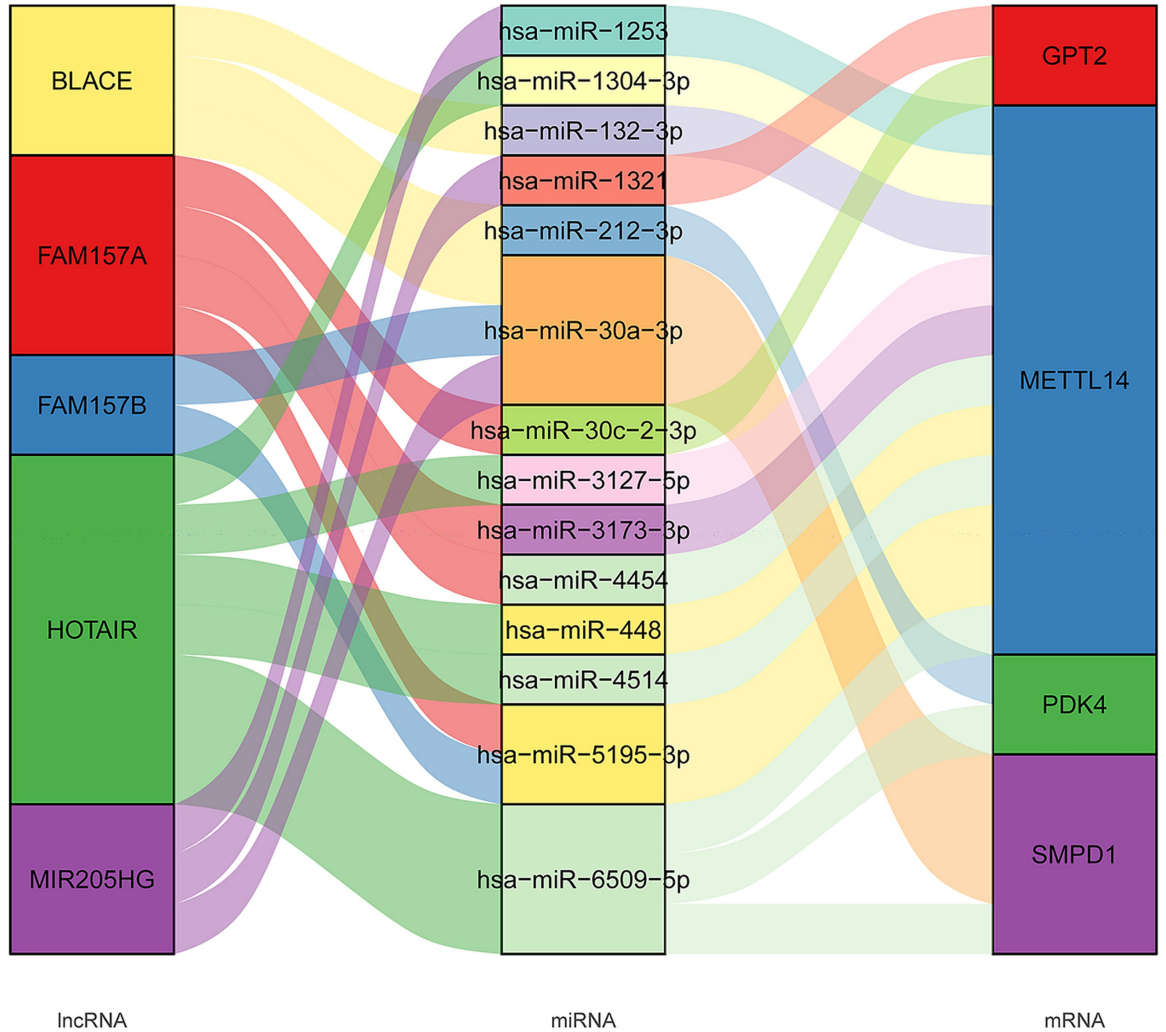
The long non-coding RNAs (lncRNAs)–microRNAs (miRNAs)–mRNAs connection relationships based on four characteristic ferroptosis related differentially expressed genes.

Furthermore, we developed a specific regulatory network focusing on four FR-DEGs used for model construction, as shown in Figure 5. From this network, we identified five lncRNAs (MIR205HG, HOTAIR, BLACE, FAM157B, and FAM157A) and 14 miRNAs (hsa-miR-1321, hsa-miR-30c-2-3p, hsa-miR-4514, hsa-miR-1253, hsa-miR-1304-3p, hsa-miR-132-3p, hsa-miR-3127-5p, hsa-miR-3173-3p, hsa-miR-4454, hsa-miR-448, hsa-miR-5195-3p, hsa-miR-6509-5p, hsa-miR-212-3p, and hsa-miR-30a-3p) that are significantly associated with the regulation of FR-DEGs. These molecules are closely associated with the regulation of sour FR-DEGs and may play critical roles in the underlying biological processes.

## 4 Discussion

In this study, we aimed to investigate novel ferroptosis-related biomarkers for TLE-HS diagnosis. Our data identified four characteristic FR-DEGs for TLE-HS, including *PDK4*, *SMPD1*, *GPT2*, and *METTL14*. Furthermore, the diagnostic model based on the four signature genes showed valuable activity, which was verified by ROC and decision curve analysis. Moreover, lncRNA–miRNA–mRNA network included five lncRNAs and 14 miRNAs. Our study identified *PDK4*, *SMPD1*, *GPT2*, and *METTL14* as novel diagnostic biomarkers in TLE-HS.

In adult humans, TLE was accepted as the most common epilepsy type, and massive neuronal loss in temporal lobe foci was the most frequently observed alteration (Zhang et al., 2024). Cai and Yang suggested that inhibition of ferroptosis could achieve neuroprotection and improve neuronal damage in epilepsy (Cai and Yang, 2021). Our data showed 47 FR-DEGs involved in six clusters were mainly enriched in apoptotic process and ferroptosis. It is well known that prolonged epilepsy would result in neuronal damage and cell death. The role of ferroptosis in neurological disorders has been widely reported. Current evidence supported that ferroptosis inhibition might be an effective therapeutic approach for epilepsy (Cai and Yang, 2021). Teocchi and D'Souza-Li showed that the progression of TLE-HS was significantly affected by three different death receptor apoptotic pathways, and they might be important targets for anti-inflammatory therapy (Teocchi and D'Souza-Li, 2016). Furthermore, we identified four characteristic FR-DEGs in TLE-HS, which might play an important role in TLE-HS. ROC of diagnostic model based on the four genes had an AUC value of 0.988 in the training dataset and 0.929 in the validation dataset, respectively, suggesting the valuable predicting role of the four genes for TLE-HS patients. Previously, Caldairou et al. (2021) showed that T2-weighted and fluid-attenuated inversion recovery/T1 features showed the highest accuracy with the AUC value of 0.95 in training cohorts and the AUC value of 0.94 in validation cohorts for revealing hippocampal pathology among TLE. Our findings might facilitate the improvement of MRI-based diagnosis for TLE-HS as an adjuvant diagnosis strategy.

It is well known that RNA m6A methylation is related to multiple kinds of neurological disorders, including epilepsy. Recent research showed a valuable role of m6A-related drugs on treating neurological disorders (Lv et al., 2023). *METTL3* and *METTL14* were critical molecules leading to RNA m6A modification (Qi et al., 2024). During the development of the nervous system, *METTL14* plays a key role in the modulation of gene expression, and its deletion could lead to disruption of cortical development by prematuring differentiation and decreasing proliferation (Li et al., 2025). Additional evidence showed knockdown *METTL14* exhibited functional axon regeneration (Wang Y. et al., 2018; Weng et al., 2018). Previous data showed that miR-1304-3p, as the regulator of *METTL14*, was decreased in TLE hippocampus and upregulated in drug-resistant serum samples (Huang et al., 2017). Moreover, previous data have confirmed the critical role of lncRNA HOTAIR on cognition and inflammation (Ahmad et al., 2024a; Ahmad et al., 2024b). The brain function impairment would be attenuated after silencing HOTAIR (Wang J. Y. et al., 2018). Meanwhile, lipopolysaccharide-induced inflammatory response and cytokine expression in macrophages were closely related with HOTAIR levels (Obaid et al., 2018). The significance of HOTAIR/miR-1304-3p/METTL14 in TLE-HS development should be further verified. SMPD1, a gene encoding acid-sphingomyelinase, generates ceramide by cleaving the phosphocholine head group of sphingomyelins. P.L302P mutated acid-sphingomyelinase triggered substrate accumulation and loss of cellular function in the central nervous system (Dodge et al., 2005). Given their roles in the nervous system, *METTL14* and *SMPD1* may impact pathophysiological and physiological brain functions.

*PDK4*, one from the PDKs family, could be widely involved in various cancers, including migration, invasion, apoptosis, and transformation (Tao et al., 2024; Yang et al., 2024). These activities were all essential for the inhibition or promotion of numerous diseases, including TLE. Furthermore, miRNA dysregulation has been widely reported in neurodegenerative disorders. Notably, miR-212-3p, as the regulator of PDK4 in TLE-HS, has also been widely reported as a regulator mediating the apoptosis and invasion of cells (Wu et al., 2020). Thus, *PDK4* might be a promising biomarker for treating TLE-HS via miR-212-39/PDK4 axis. GPT is an alanine transaminase leading to the generation of pyruvate and glutamate by catalyzing the reversible transamination between *α*-ketoglutarate and alanine. High levels of GPT2 mediate the proliferation of various tumor cells, which is important for tumor growth (Cao et al., 2017; Yao et al., 2025). Although there is relatively limited research regarding its connection with epilepsy, it is recommended to verify further the roles of *GPT2* and miR-212-39/PDK4 in TLE.

Our study has several limitations. First, despite utilizing multiple GEO datasets to identify potential ferroptosis-related gene signatures for TLE-HS, the sample size remained limited. Larger cohort studies are necessary to validate our findings. Second, we did not explicitly explore the mechanistic interactions between ferroptosis and other critical pathways involved in epilepsy pathogenesis—such as hypoxia signaling and neuroinflammatory cascades—due to our primary focus on constructing a diagnostic model. Finally, although the bioinformatic predictions suggested potential ceRNA regulatory networks, experimental validation using *in vitro* seizure models or patient-derived tissues is required to confirm these interactions.

Therefore, studying the role of *PDK4*, *SMPD1*, *GPT2*, and *METTL14* in regulating the pathogenesis of central nervous system, brain injury, and cell activities may help improve diagnostic strategies for TLE-HS. It might have great potential application in the clinical practice of TLE-HS. However, our study should note some limitations, considering the individual heterogeneity and the limited number of enrolled subjects. Our findings should be further validated using more multicenter clinical data.

## Data Availability

The original contributions presented in the study are included in the article/Supplementary material, further inquiries can be directed to the corresponding author.

## Footnotes

^1^ https://bioconductor.org/packages/release/bioc/html/limma.html

^2^ http://www.datjar.com:40013/bt2104/

^3^ https://david.ncifcrf.gov/

^4^ https://cran.r-project.org/web/packages/WGCNA/index.html

^5^ http://carolina.imis.athena-innovation.gr/diana_tools/web/index.php

^6^ http://129.206.7.150/

^7^ http://www.cytoscape.org/

^8^ https://cran.r-project.org/web/packages/rms/index.html

^9^ https://cran.r-project.org/web/packages/lars/index.html

^10^ https://cran.r-project.org/web/packages/e1071

^11^ https://cran.r-project.org/web/packages/pROC/index.html

^12^ https://cran.r-project.org/web/packages/rmda/index.html
