# Supplementary figures and images for: Identification of ferroptosis-related gene signatures in temporal lobe epilepsy with hippocampal sclerosis
Source: Front Neurosci. 2025 Apr 2;19:1530182. doi: 10.3389/fnins.2025.1530182 (PMC11999976; doi:10.3389/fnins.2025.1530182)

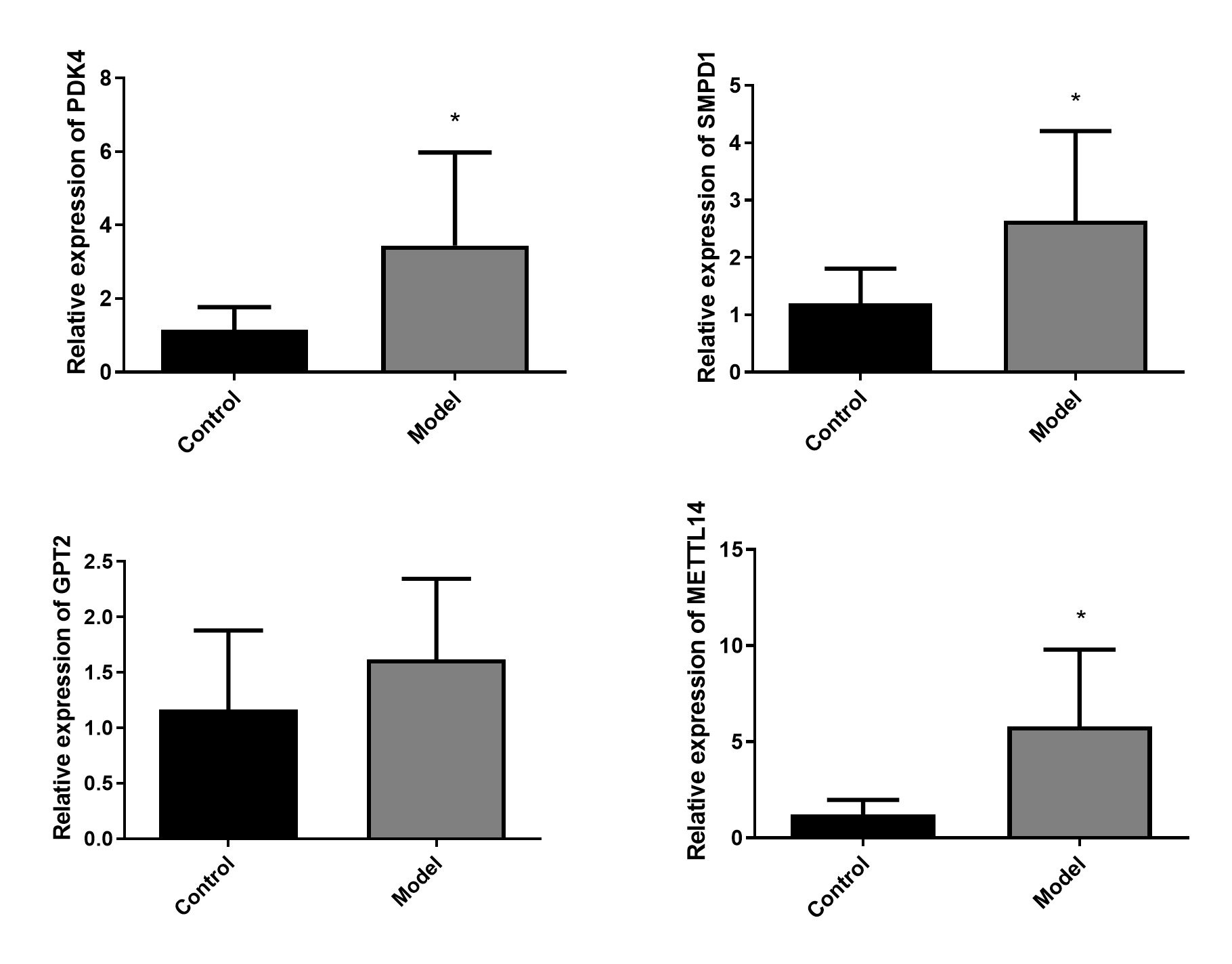

Supplement: Supplementary file 1 [file Image_1.tif]
